# Supplementary material for: Altermagnetic lifting of Kramers spin degeneracy
Source: Nature. 2024 Feb 14;626(7999):517–22. doi: 10.1038/s41586-023-06907-7 (PMC10866710; doi:10.1038/s41586-023-06907-7)
Supplement: Supplementary file 1 — This file contains experimental figures (Supplementary Figs. 1–11), supplementary text describing characterization of bulk MnTe samples and supplementary references. [file 41586_2023_6907_MOESM1_ESM.pdf]

nature portfolio

https://doi.org/10.1038/s41586-023-06907-7

Supplementary information

Altermagnetic lifting of Kramers spin degeneracy

In the format provided by the authors and unedited

Supplementary information:
Altermagnetic lifting of Kramers spin degeneracy

J. Krempaský,[1,*] L. Šmejkal,[2,3,*] S. W. D'Souza,[4,*] M. Hajlaoui,[5] G. Springholz,[5] K. Uhlířová,[6] F. Alarab,[1] P. C. Constantinou,[1] V. Strokov,[1] D. Usanov,[1] W. R. Pudelko,[1] R. González-Hernández,[7] A. Birk Hellenes,[2] Z. Jansa,[4] H. Reichlová,[3] Z. Šobáň,[3] R. D. Gonzalez Betancourt,[3] P. Wadley,[8] J. Sinova,[2,3] D. Kriegner,[3] J. Minár,[4,†] J. H. Dil,[9,1] and T. Jungwirth[3,8,†]

[1]*Photon Science Division, Paul Scherrer Institut, CH-5232 Villigen, Switzerland*

[2]*Institut für Physik, Johannes Gutenberg Universität Mainz, D-55099 Mainz, Germany*

[3]*Institute of Physics, Czech Academy of Sciences, Cukrovarnická 10, 162 00 Praha 6 Czech Republic*

[4]*University of West Bohemia, New Technologies Research Center, Plzen 30100, Czech Republic*

[5]*Institute of Semiconductor and Solid State Physics, Johannes Kepler University Linz, 4040 Linz, Austria*

[6]*Faculty of Mathematics and Physics, Charles University, Ke Karlovu 3, CZ-121 16, Prague 2, Czech Republic*

[7]*Grupo de Investigación en Física Aplicada, Departamento de Física, Universidad del Norte, Barranquilla, Colombia*

[8]*School of Physics and Astronomy, University of Nottingham, Nottingham NG7 2RD, United Kingdom*

[9]*Institut de Physique, École Polytechnique Fédérale de Lausanne, CH-1015 Lausanne, Switzerland*

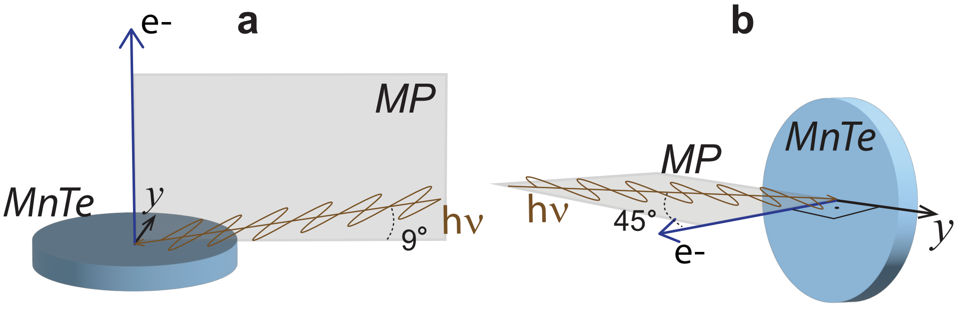

Fig. S 1. **Geometry of photoemission experiments.** **(a)** Soft X-ray ARPES experiment at ADRESS beamline corresponding to the normal emission. The X-rays come to the sample at a grazing-incidence angle of 9°. The analyzer lens axis is perpendicular to the sample surface, the incident π-polarized beam and analyzer lens form the vertical measurement plane (MP). **(b)** Normal emission geometry of the ultraviolet spin-polarized ARPES (UV SARPES) experiment at the COPHEE beamline with incident π-polarized beam in the horizontal MP. The y-axes indicate the sample direction along the y-component of the spin expectation values.

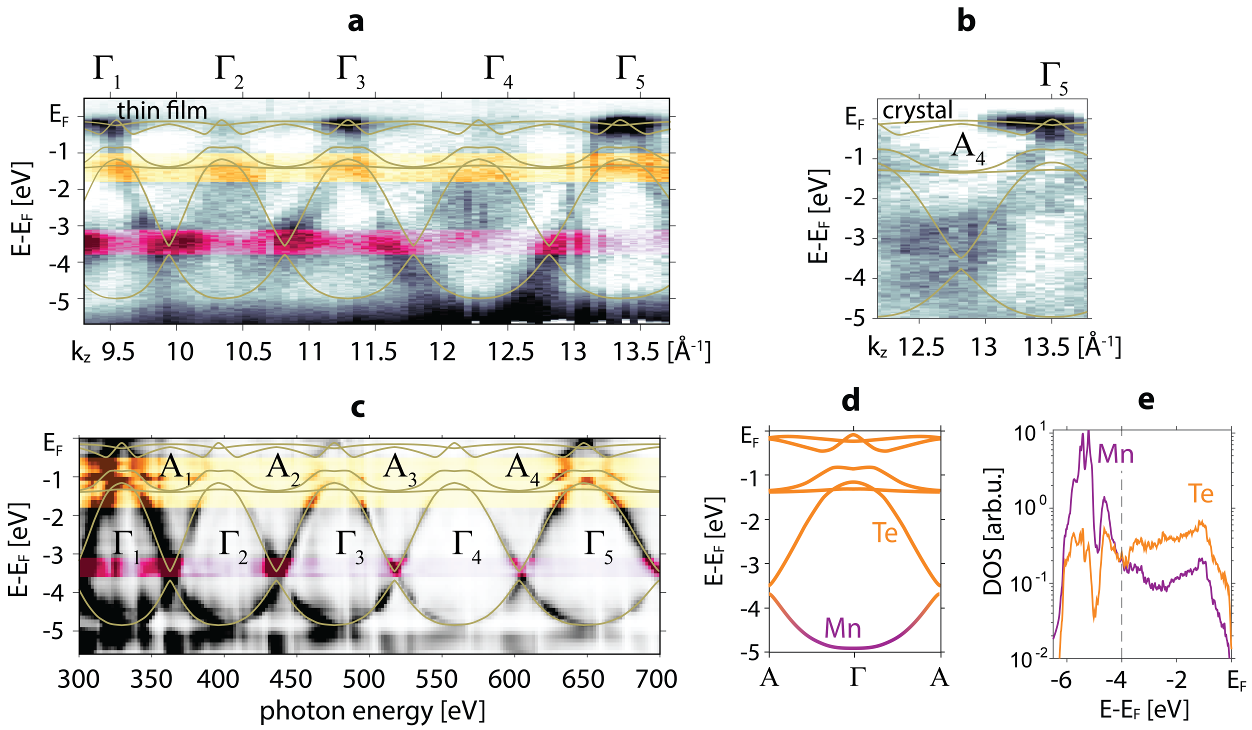

Fig. S 2. **MnTe electronic structure under normal emission k_z-scan with photon energy**. **(a)** Experimental k_z-scan band map from MnTe thin film over five Brillouin zones Γ_{1-5}, corresponding to a 300–700 eV photon energy scan. **(b)** k_z-scan band map from MnTe cleaved bulk-crystal around Γ_5. **(c)** Corresponding one-step calculations. The colored stripes highlight surface resonances as partially localized Te-states (yellow shades) and partially localized Mn-states (purple shades), **(d)** Bloch spectral function calculations (BSF) along the $\mathbf{A}-\mathbf{\Gamma}-\mathbf{A}$ k_z-direction. **(e)** Density of states (DOS) projections of the BSF atomic orbital projections from Te p-orbitals (yellow lines) compared to Mn d-orbitals (purple lines). The photon energy dependent data were used to find the $k_z = 0$ plane for identifying the weak altermagnetic lifting of Kramers spin degeneracy, and optimal photon energies to observe the strong altermagnetic lifting of Kramers spin degeneracy at $k_z \neq 0$. As MnTe possess two magnetic sublattices connected by a sixfold screw axis along [0001][1], the spectral weight in such a non-symmorphic symmetry is suppressed in the even Brillouin zones ($\mathbf{\Gamma}_2$ and $\mathbf{\Gamma}_4$)[2]. Band structure calculations are overlaid on both experimental data (panels **a,d**) and one-step photoemission calculations (panel **c**).

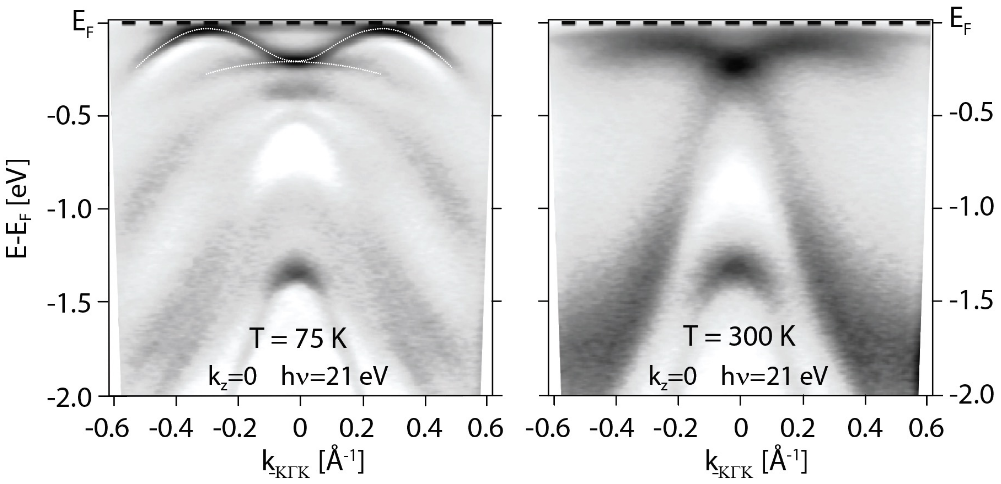

Fig. S 3. **Comparison of low-temperature (left) and high-temperature (right) UV ARPES measurements.** Measurements were performed at photon energy 21 eV corresponding to $k_z = 0$. Splitting of the top two bands at low temperature, consistent with the splitting observed by soft X-ray ARPES (Figs. 2,3 in the main text), is highlighted by white dotted lines. The experiments were performed on the UV ARPES beamline URANOS at SOLARIS synchrotron facility. Samples used in these measurements are MnTe thin films grown by molecular-beam epitaxy. We used a vacuum suitcase to transfer the thin-film samples from the growth to the UV ARPES chamber without breaking ultra-high-vacuum conditions.

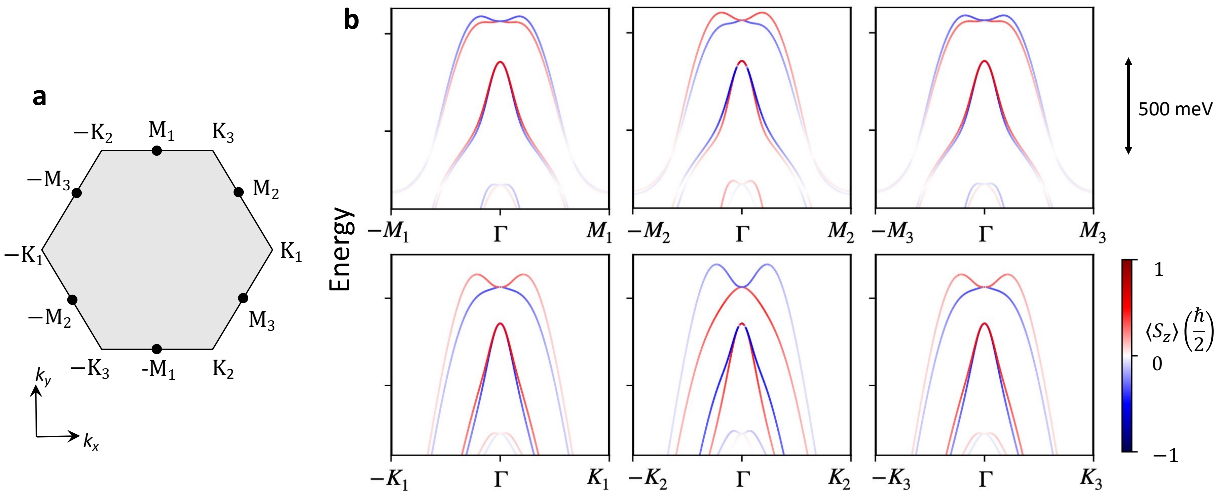

Fig. S 4. *Ab initio* spin-polarized energy bands of MnTe showing weak altermagnetic spin splitting calculated by by VASP (see Methods). **(a)** shows the $k_z = 0$ nodal plane with notation of $\mathbf{K}_i$, $\mathbf{M}_i$ points. **(b)** shows the spin-polarized energy bands along the $\mathbf{\Gamma} - \mathbf{K}_i$, $\mathbf{\Gamma} - \mathbf{M}_i$ paths in this plane. There are two $\mathbf{\Gamma} - \mathbf{M}_i(\mathbf{K}_i)$ paths showing the same splitting magnitude and one with a different magnitude. This follows from symmetry when the Néel vector is along the high-symmetry easy axis corresponding to one of the $\mathbf{\Gamma} - \mathbf{M}_i$ directions (the Néel-vector axis corresponds to $\mathbf{\Gamma} - \mathbf{M}_2$ in the presented calculations).

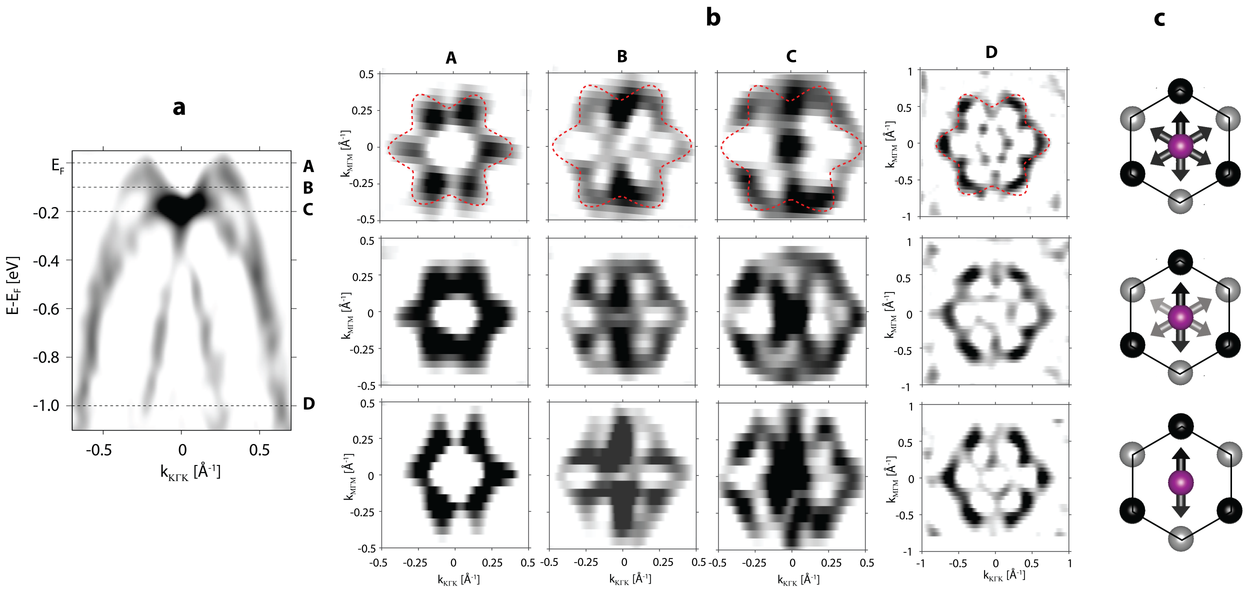

Fig. S 5. **Experimental constant-energy maps and Néel-vector easy-axis domains**. **(a)** $\mathbf{\Gamma - K}$ band map from Fig. 3c of the main text with the indicated binding energies A-D. **(b)** Experimental constant energy maps recorded at three different sample locations (top, middle and bottom row) with different domain distributions. **(c)** Related schematics of the Néel-vector easy axes. Dashed red contours in top row highlight the 6-fold symmetry for all the constant-energy cuts A-D for the top-row sample location. The 2-fold anisotropy in the middle and bottom rows is clearer close to the top of the valence band (A,B cuts) and can be used as a measure for the presence of the prevailing Néel vector easy-axis.

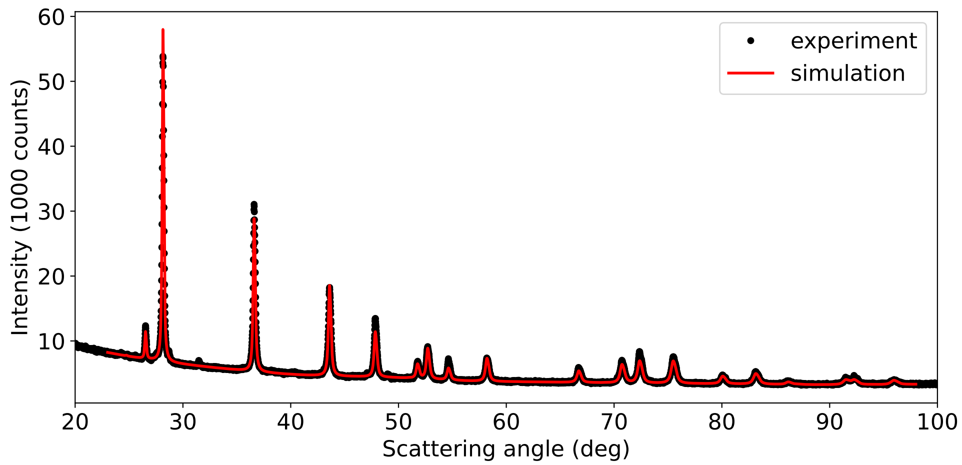

Fig. S 6. **X-ray powder diffraction characterization of powder-milled MnTe single crystals.** Experimental data are shown together with a simulation of the α-MnTe powder diffraction pattern. A small peak at 31.5 deg which is not accounted for in the simulation originates from a $MnTe_2$ impurity phase.

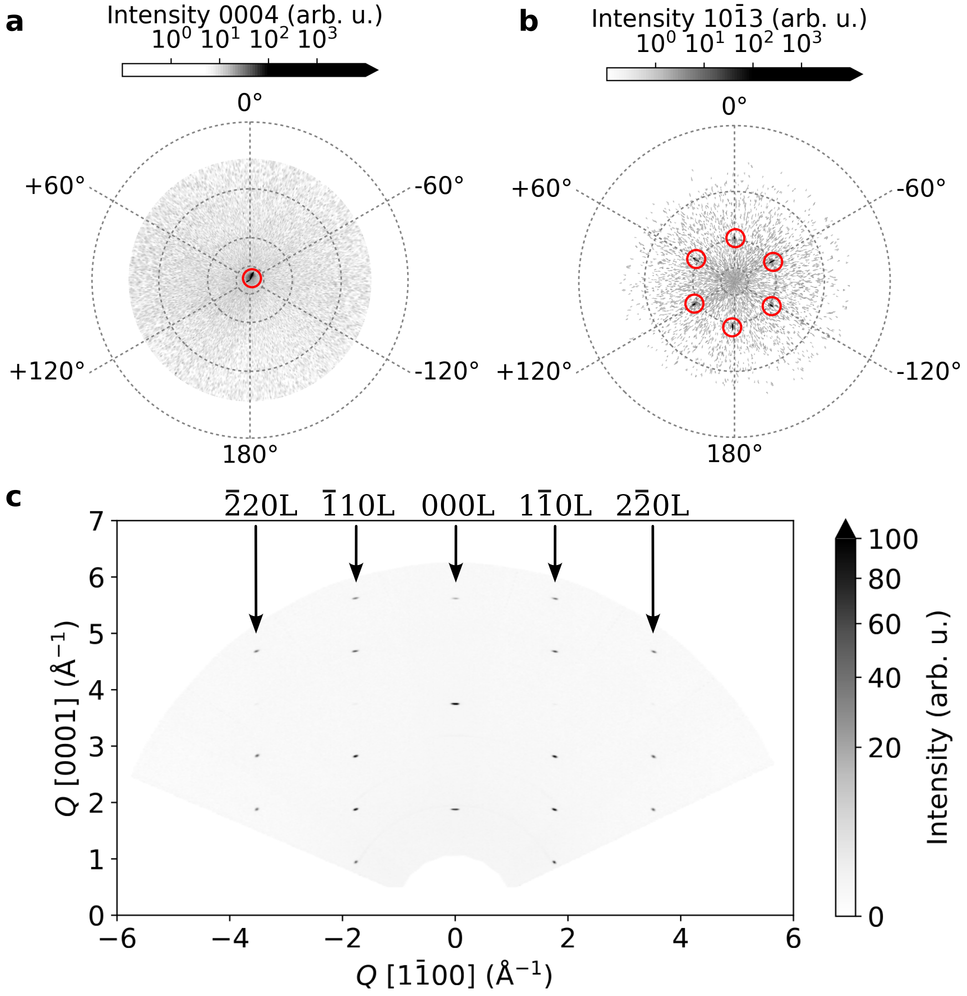

Fig. S 7. **X-ray diffraction characterization of MnTe single crystals.** **(a)** and **(b)** show pole figure measurements of the MnTe 0004 and $10\bar{1}3$ lattice planes, respectively. Red circles mark the expected peak positions for an α-MnTe single crystal. **(c)** Reciprocal space map measurement of the [$1\bar{1}00$] / [0001] diffraction plane showing various series of diffraction peaks whos Bragg indices are indicated in the panel.

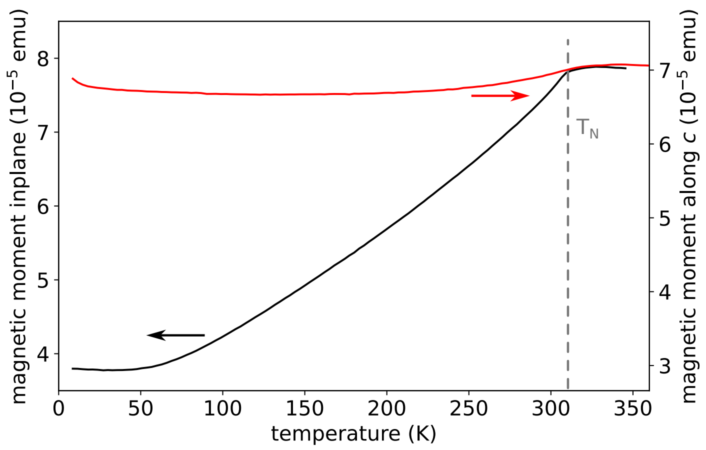

Fig. S 8. **Temperature dependent magnetic susceptibility.** Magnetic field is applied within (black curve) and perpendicular (red curve) to the hexagonal basal plane. The Néel Temperature of 309 K is indicated in the figure and marks the point were the susceptibility upon cooling drops strongly for field within the basal plane whereas it stays nearly constant for magnetic field along the *c*-axis. Different offsets of the inplane and out of plane data originate from the different backgrounds of the sample holder for the different mounting orientations.

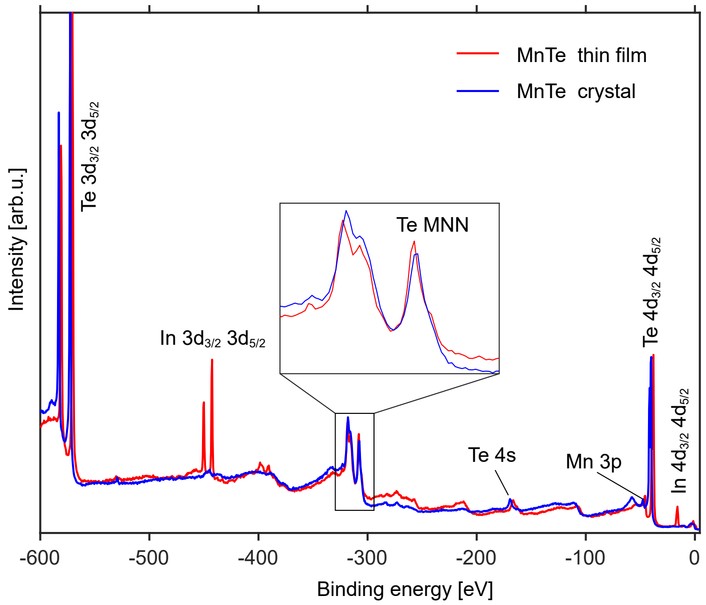

Fig. S 9. **X-ray photoelectron core level spectroscopy (XPS) of the (0001) surface**. XPS of a MnTe thin film grown on InP substrate (red line) and cleaved bulk-crystal (blue line). The inset compares the spectra of the Te MNN Auger peaks.

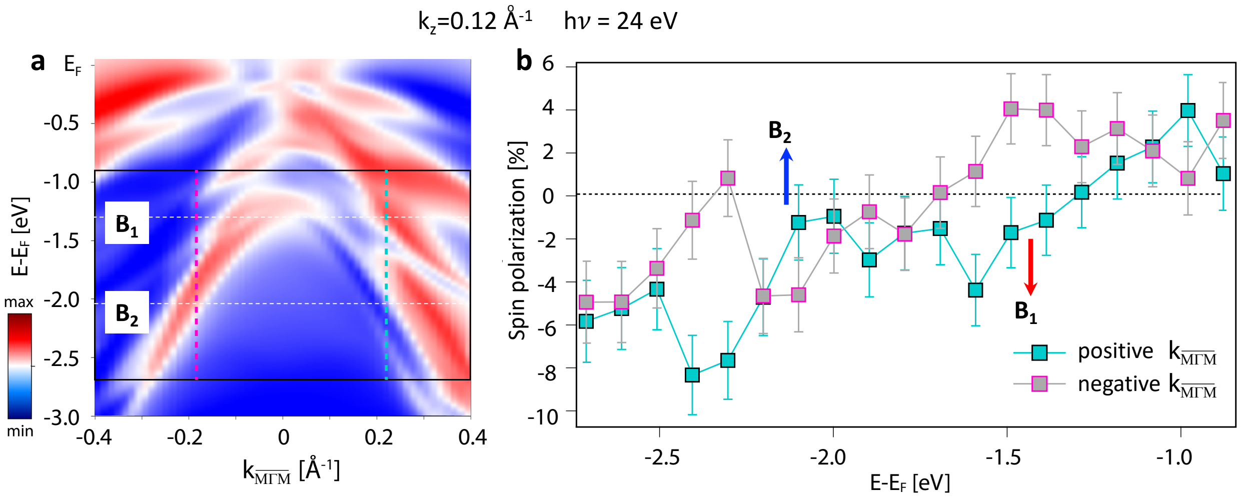

Fig. S 10. **Spin resolved ARPES simulations and measurements. (a)** One-step SARPES simulations. Red and blue colors show opposite spin-polarization components along the Néel vector, corresponding to the $\mathbf{\Gamma - M}$ axis. **(b)** SARPES signals for positive and negative values of the momentum component along the $\bar{\mathbf{\Gamma}} - \bar{\mathbf{M}}$ path, highlighted by dashed lines in (a). (Data for the negative component are replotted from Fig. 4g of the main text.) The spin polarization component is detected along an axis corresponding to the $\mathbf{\Gamma - M}$ direction.

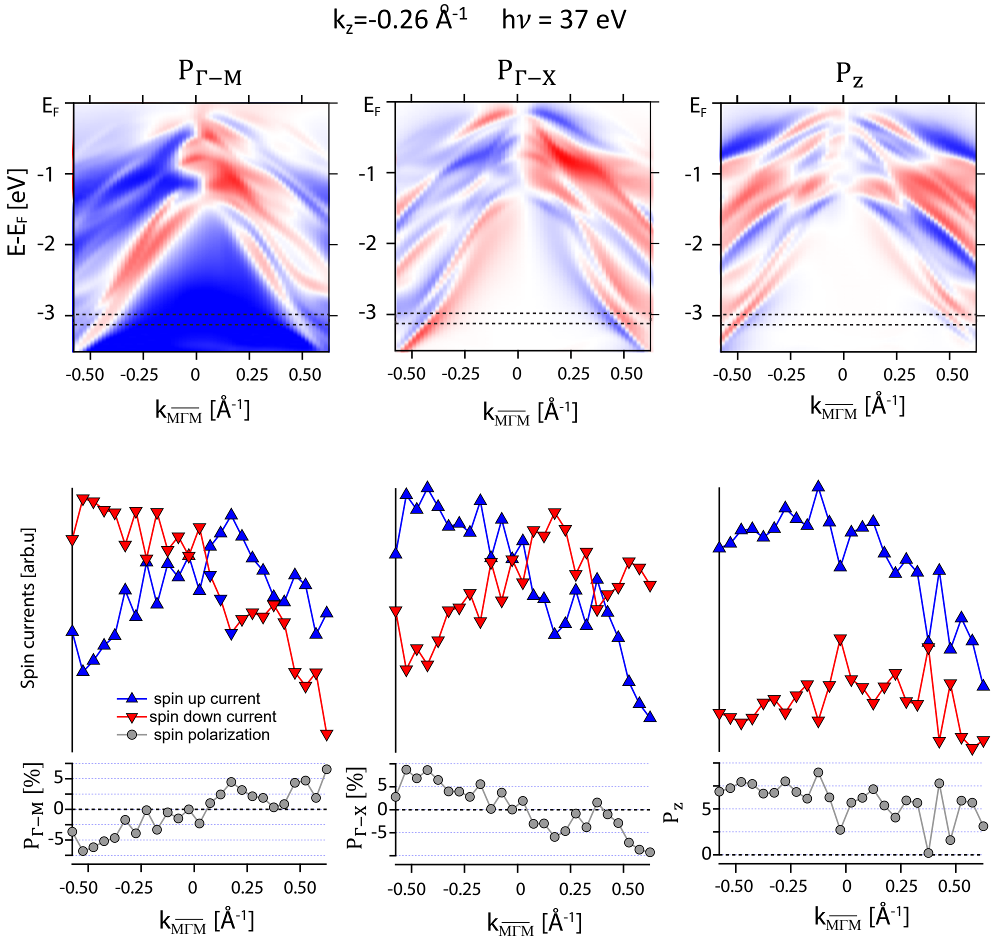

Fig. S 11. **Spin resolved ARPES simulations (top) and measurements (bottom) for the three orthogonal spin-polarization components.** Consistent with the one-step SARPES simulations, the measured SARPES data show a prevailing antisymmetric dependence on momentum of the in-plane spin-polarization components (left and middle panels) along the selected momentum-distribution curve (MDC), corresponding to the dashed frame in the theoretical panels. The out-of-plane spin-polarization component (right panel) shows a prevailing symmetric dependence, again consistent with the simulations.

Characterization of bulk MnTe samples

Structural characterization by X-ray diffraction

Our α-MnTe bulk samples crystallize in plate-like single crystals with the NiAs structure and lattice parameters of $a = 4.149(1)$ Å and $c = 6.715(1)$ Å. The lattice parameters are within the range of values reported for MnTe in literature[3–6] and were obtained from the powder diffraction measurement in Fig. S 6. The single crystalline platelets have their c-axis perpendicular to the typically several mm^2 large planes. X-ray diffraction measurements illuminating ~ 2 mm^2 of the crystals indicate single crystalline orientation throughout the probed sample volume and are shown in Fig. S 7. X-ray diffraction pole figure measurements of the 0004 and $10\bar{1}3$ lattice planes indicate the perfect crystalline orientation within a several mm^2 object. Reciprocal space map measurements corroborate these results and in addition show the characteristic peak intensity variations and extinctions related to the α-MnTe structure. X-ray characterization of the epitaxial layers evidence a perfect expitaxial relationship to the InP(111) substrates with the c-axis perpendicular to the surface. Lattice parameters as a function of temperature have been reported in Refs.[7–9].

Magnetometry

SQUID magnetometry measurements in Fig. S 8 were performed in order to confirm the compensated magnetic order and magnetic anisotropy. In agreement with susceptibility measurements in Refs.[9,10] we find that the susceptibility drops considerable below the Néel temperature $T_N = 309$ K for magnetic field within the basal plane. This indicates that the magnetic moments are oriented in the hexagonal basal plane in agreement with existing neutron diffraction data[9,11].

X-ray photoelectron spectroscopy

Fig. S 9 shows X-ray photoelectron spectroscopy (XPS) data measured at a photon energy of 800 eV with π-polarized light measured at the ADRESS experimental station (see Methods). The indicated Te core-levels are without oxidation satellite features, indicating that the thin-film and cleaved bulk-crystal surfaces reflect pristine MnTe without oxidation and

degradation. The survey spectra from MnTe thin films indicate presence of highly diffusive Indium atoms from the InP(001) substrate on top of which MnTe epilayers are grown. The absence of satellites in the Te core levels indicates that these In atoms are present as amorphous clusters and will not contribute to the coherent spectral weight in our ARPES data. The inset in Fig. S 9 compares the spectra of the Te MNN Auger peaks carrying information about the chemical state of the Te-atoms. Their peak shapes and locations further confirm the equivalent chemical composition between MnTe thin films and bulk-crystals. Hence, the ARPES data in the main text reflect intrinsic MnTe properties from both thin films and crystals.

---

[*] These three authors contributed equally

[†] Corresponding authors: juraj.krempasky@psi.ch, jminar@ntc.zcu.cz, jungw@fzu.cz

[1] R. D. Gonzalez Betancourt, J. Zubáč, R. Gonzalez-Hernandez, K. Geishendorf, Z. Šobáň, G. Springholz, K. Olejník, L. Šmejkal, J. Sinova, T. Jungwirth, S. T. B. Goennenwein, A. Thomas, H. Reichlová, J. Železný, and D. Kriegner, Phys. Rev. Lett. **130**, 036702 (2023).

[2] G. Landolt, S. V. Eremeev, O. E. Tereshchenko, S. Muff, B. Slomski, K. A. Kokh, M. Kobayashi, T. Schmitt, V. N. Strocov, J. Osterwalder, *et al.*, New Journal of Physics **15**, 085022 (2013).

[3] S. Greenwald, Acta Crystallographica **6**, 396 (1953).

[4] T. Tien, R. Martin, and L. Van Vlack, Transactions of the Metallurgical Society of AIME **242**, 2153 (1968).

[5] F. Grønvold, N. J. Kveseth, F. D. S. Marques, and J. Tichy, The Journal of Chemical Thermodynamics **4**, 795 (1972).

[6] G. I. Makovetskii, A. I. Galyas, G. M. Severin, and K. I. Yanushkevich, ChemInform **28** (1997), 10.1002/chin.199704029.

[7] D. Kriegner, E. Wintersberger, and J. Stangl, Journal of Applied Crystallography **46**, 1162 (2013).

[8] A. Damascelli, Z. Hussain, and Z.-X. Shen, Rev. Mod. Phys. **75**, 473 (2003).

[9] D. Kriegner, H. Reichlova, J. Grenzer, W. Schmidt, E. Ressouche, J. Godinho, T. Wagner, S. Y. Martin, A. B. Shick, V. V. Volobuev, G. Springholz, V. Holý, J. Wunderlich, T. Jungwirth, and K. Výborný, Physical Review B **96**, 214418 (2017).

[10] T. Komatsubara, M. Murakami, and E. Hirahara, Journal of the Physical Society of Japan **18**, 356 (1963).

[11] N. Kunitomi, Y. Hamaguchi, and S. Anzai, Journal de Physique **25**, 568 (1964).
